# Damage and Failure Monitoring of Aerospace Insulation Layers Based on Embedded Fiber Bragg Grating Sensors

**DOI:** 10.3390/polym16243543

**Published:** 2024-12-19

**Authors:** Guang Yan, Boli Wan, Heng Huang, Wuyi Li

**Affiliations:** 1 College of Instrument Science and Opto-Electronics Engineering, Beijing Information Science & Technology University, Beijing 100101, China; yanguang79@bistu.edu.cn (G.Y.);; 2 Research Institute of Aero-Engine, Beijing University of Aeronautics and Astronautics, Beijing 102206, China

**Keywords:** carbon fiber-reinforced composites, fiber Bragg grating, finite element analysis, damage evolution, structural health monitoring

## Abstract

Carbon fiber-reinforced polymer (CFRP) composites are widely used in aviation thermal insulation layers due to their high strength-to-weight ratio and excellent high-temperature performance. However, challenges remain regarding their structural integrity and durability under extreme conditions. This study first employed finite element simulation to model the damage evolution of CFRP laminated plates under axial tensile loads and their thermal decomposition behavior in high-temperature environments, providing a theoretical reference. Subsequently, experimental research was conducted on CFRP laminated plates embedded with fiber Bragg grating (FBG) sensors. In the tensile tests, FBG sensors accurately monitored the entire process from elastic deformation to damage propagation and eventual failure. In the high-temperature tests, despite a 75% reduction in tensile strength, FBG sensors effectively monitored damage evolution. Conclusively, the results demonstrate that FBG sensors possess reliable monitoring capabilities under complex conditions, making them a promising solution for the long-term structural health monitoring of aviation thermal insulation materials and paving the way for future developments in this area.

## 1. Introduction

Aviation thermal insulation layers are crucial in the field of aerospace, protecting aircraft structures from extreme environments such as high temperatures and pressures. These layers effectively block heat and prevent the overheating of internal structures and critical equipment, ensuring safe and efficient flights during complex missions and the secure return of spacecraft. However, during flight, external loads such as vibrations, impacts, aerodynamic pressure, and thermal expansion/contraction cycles can cause material fatigue, stress concentration, and microcracking. High-temperature environments, especially during high-speed flight or atmospheric re-entry, accelerate material aging, leading to thermal stress concentration and thermal fatigue [1,2,3]. This damage gradually expands within the insulation layer, degrading its thermal insulation performance and structural integrity, ultimately compromising the safety of the aircraft.

Therefore, the structural health monitoring (SHM) of aviation thermal insulation layers to detect and repair damage in a timely manner is a pressing issue. Carbon fiber-reinforced polymer (CFRP) composites have been widely used in aviation thermal insulation layers due to their high strength, light weight, and corrosion resistance [4,5,6]. This paper focuses on CFRP laminated plates to study the structural health during the service life of insulation layers.

In recent years, fiber optic sensing technology, as a non-invasive, real-time monitoring method, has been increasingly applied in the aerospace field for damage detection [7,8]. Among them, fiber Bragg grating (FBG) sensors have become a research hotspot in damage monitoring due to their high sensitivity, resolution, and resistance to electromagnetic interference [9,10,11]. Combining FBG sensors with CFRP to create smart composites has shown strong potential in applications such as wind turbine blades and aerospace, where sensor size and weight are critical.

S. Takeda et al. [12] combined finite element analysis (FEA) with FBG sensors, embedding small-diameter FBG sensors into CFRP composite structures. By analyzing the changes in the reflected spectrum, they monitored damage such as delamination and matrix cracking under tensile load. The results showed that as the number of matrix cracks increased, the reflected spectrum width of the FBG sensor also increased, and the results were validated by FEA. Shin et al. [13] embedded FBG sensors into single-layer lap joints of composites and conducted tensile and fatigue loading experiments. The damage initiation and propagation were reflected by the peak splitting and broadening of the full spectrum response of the FBG. Zhu et al. [14] used FBG sensors to detect internal damage in composite samples under low-frequency cycling. The results showed that when the FBG sensor was 15 mm away from the damage and the damage size was below 1.5 mm, the damage could not be accurately identified. Under low-frequency cycling, when the FBG sensor was 15 mm away from the damage and the damage size exceeded 2 mm, the damage could be identified. Yin et al. [15] combined finite element analysis with FBG sensors and proposed a strain-based structural damage identification model. The experimental results showed that the model could accurately identify hole damage positions with an accuracy rate of over 98%, and crack direction recognition accuracy was over 91%. ZHAN et al. [16] encapsulated FBG in a capillary ceramic tube to create a strain-free temperature sensor, embedding it into composites to monitor the temperature and strain during the autoclave curing process. By comparing the temperature monitoring results of ordinary gratings, high-temperature gratings, and thermocouples, they verified the accuracy of temperature monitoring, demonstrating that FBG can monitor the out-of-autoclave curing process of composites and obtain internal temperature and strain data, improving the quality of thermoset composite products. Ma et al. [17] proposed a tilted fiber Bragg grating sensor to monitor the curing of CFRP composite materials. The experimental results show that the sensor is very sensitive to the curing residual strain of CFRP and can be used in applications involving composite curing processes.

These studies show that embedded fiber Bragg grating sensor technology has made significant progress in monitoring the damage and curing process of carbon fiber-reinforced polymer structures. However, there remains a gap in understanding the performance of FBG sensors in complex working conditions. This paper takes the CFRP laminates used as aviation thermal insulation materials as the research object, uses embedded FBG sensors for monitoring, and studies the evolution of the material from intact state to damage failure under the influence of external loads and high-temperature environments. The response characteristics of FBG sensors under complex working conditions are explored, and the mechanical response of CFRP laminates in complex stress environments is observed, including stress–strain relationships, changes in strength and stiffness, and fatigue and damage evolution of materials.

## 2. Principle

### 2.1. Fiber Bragg Grating Sensor Principle

A fiber Bragg grating is created by exposing the core of a single-mode optical fiber transversely to strong ultraviolet light with a periodic pattern. The exposure to UV light permanently increases the refractive index of the fiber core, creating a fixed refractive index modulation based on the exposure pattern, known as the grating.

At each periodic refractive index change, a small amount of light is reflected. When the grating period is approximately half the wavelength of the incident light, all reflected light coherently combines into a single beam of highly reflective light. This is known as the Bragg condition. The wavelength of the reflected light is referred to as the Bragg wavelength. Other wavelengths of light signals are almost unaffected by the Bragg grating and continue to transmit through the fiber. The Bragg wavelength condition is expressed as

(1) $$\lambda_{B}={\;2n}_{\mathrm{eff}}\Lambda$$

where $\lambda_{B}$ is the Bragg wavelength, $n_{\mathrm{eff}}$ is the effective refractive index of the fiber core, and Λ is the grating period.

When the FBG sensor is affected by strain or temperature, the Bragg wavelength shifts as shown in Equation (2):

(2) $$\frac{\Delta \lambda_{B}}{\lambda_{B}}=(1-P_{e})\Delta \varepsilon +(\alpha +\xi )\Delta T$$

where $\Delta \lambda_{B}$ is the Bragg wavelength shift, Δε is the strain variation, $P_{e}$ is the effective photoelastic coefficient, α is the thermal expansion coefficient, and ξ is the thermo-optic coefficient. According to Equation (2), if the experiment is conducted at a constant temperature or temperature compensation is applied, the relationship between the Bragg wavelength shift and the strain variation is expressed as

(3) $$\frac{\Delta \lambda_{B}}{\lambda_{B}}=(1-P_{e})\Delta \varepsilon$$

Since $P_{e}$ is a constant, it can be deduced from Equation (3) that under conditions without temperature influence, the Bragg wavelength shift and strain variation are linearly related. Thus, FBG can serve as a strain sensing unit. When both temperature and mechanical strain cause a shift in the FBG sensor’s Bragg wavelength, to accurately measure strain using the FBG sensor, temperature compensation is necessary to decouple the strain value.

### 2.2. Failure Criteria for CFRP Laminates

This study adopts the 3D Hashin failure criterion [18] and develops the corresponding Abaqus subroutine to determine whether damage occurs in composite laminates and to identify the damage modes. The expressions for the 3D Hashin failure criterion are as follows:

1. Fiber tensile failure ($\sigma_{11}$ > 0)
  (4) $$F_{ft}={\left(\frac{\sigma_{11}}{X_{t}}\right)}^{2}+{\left(\frac{\tau_{12}}{S_{12}}\right)}^{2}+{\left(\frac{\tau_{13}}{S_{13}}\right)}^{2}\geq 1$$
2. Fiber compression failure ($\sigma_{11}$ < 0)
  (5) $$F_{fc}={\left(\frac{\sigma_{11}}{X_{c}}\right)}^{2}\geq 1$$
3. Matrix tensile failure ($\sigma_{22}$ > 0)
  (6) $$F_{mt}={\left(\frac{\sigma_{22}}{Y_{t}}\right)}^{2}+{\left(\frac{\tau_{12}}{S_{12}}\right)}^{2}+{\left(\frac{\tau_{13}}{S_{13}}\right)}^{2}\geq 1$$
4. Substrate compression failure ($\sigma_{22}$ < 0)
  (7) $$F_{mc}={\left(\frac{\sigma_{22}}{Y_{c}}\right)}^{2}+{\left(\frac{\tau_{12}}{S_{12}}\right)}^{2}+{\left(\frac{\tau_{23}}{S_{23}}\right)}^{2}\geq 1$$
5. Tensile delamination failure (σ_33_ > 0):
  (8) $$F_{dlt}={\left(\frac{\sigma_{33}}{Z_{\iota }}\right)}^{2}+{\left(\frac{\sigma_{13}}{S_{13}}\right)}^{2}+{\left(\frac{\sigma_{23}}{S_{23}}\right)}^{2}\geq 1$$
6. Compressive delamination failure (σ_33_ < 0):
  (9) $$F_{dlc}={\left(\frac{\sigma_{33}}{Z_{c}}\right)}^{2}+{\left(\frac{\sigma_{13}}{S_{13}}\right)}^{2}+{\left(\frac{\sigma_{23}}{S_{23}}\right)}^{2}\geq 1$$
  where $\sigma_{11}$, $\sigma_{22}$, and $\sigma_{33}$ are positive stresses; $\tau_{12}$, $\tau_{13}$, and $\tau_{23}$ are shear stresses; $X_{t}$ and $X_{c}$ are the longitudinal tensile and compressive strengths of the single-layer plate; $Y_{t}$ and $Y_{c}$ are the transverse tensile and compressive strengths of the single-layer plate; $Z_{t}$ and *$Z_{c}$* are the tensile and compressive strengths perpendicular to the direction of the single-layer plate; and $S_{12}$, $S_{13}$, and $S_{23}$ are the longitudinal and the two transverse shear strengths, respectively.

After satisfying the failure criteria, the damage variables representing the remaining elastic modulus of the damaged material are used to describe the constitutive relationships of the composite material. The overall damage states of the fibers and matrix are represented by the damage variables $d_{f}$ and $d_{m}$, respectively [18].

(10) $$d_{f}=1-(1-d_{ft})(1-d_{\mathrm{fc}})$$

(11) $$d_{m}=1-(1-d_{\mathrm{mt}})(1-d_{\mathrm{mc}})$$

When $F_{ft}$ ≥ 1, $d_{ft}$ = 1; $F_{fc}$ ≥ 1, $d_{fc}$ = 1; when $F_{mt}$ ≥ 1, $d_{mt}$ = 1; $F_{mc}$ ≥ 1, $d_{mc}$ = 1.

## 3. Finite Element Simulation Analysis

### 3.1. Finite Element Simulation of CFRP Laminated Plate Strength

**Figure 1:**
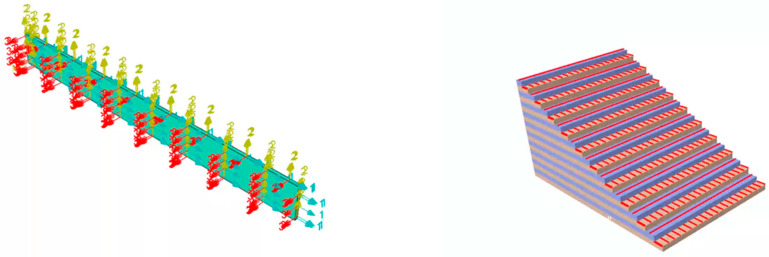
Lamination sequence of CFRP laminated plate.

**Figure 2:**
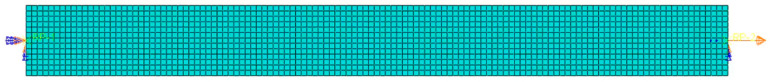
Schematic diagram of boundary conditions of the specimen.

To explore the structural changes in aviation thermal insulation materials under axial loads, a 3D Hashin failure criterion is used to simulate the tensile failure process of CFRP laminated plates. In ABAQUS 2023 software, a 3D solid structure with dimensions of 250 × 25 × 2.5 mm is created. The selected element type is C3D8R, and the mesh is generated from the top to the bottom in the thickness direction. The lamination sequence is alternated at 0°/90°, with each single-layer plate thickness set to 0.125 mm, and a total of 20 layers are used for the specimen, with an overall thickness of 2.5 mm. The lamination sequence of the test specimen is shown in Figure 1. The material parameters used are shown in Table 1. The mesh division result is shown in Figure 2.

The material properties are defined using the UserMaterial option in the ABAQUS material definition module. The material density, elastic parameters, and 3D Hashin damage criterion parameters are assigned. A displacement load of 10 mm is applied at the right end of the CFRP laminated plate. In the output manager, configuring and extracting the stress, strain, and damage variables for each layer of the CFRP laminate is essential for analyzing the stress response and damage evolution of the material under loading conditions.

### 3.2. Tensile Load Damage Study of the Model

**Figure 3:**
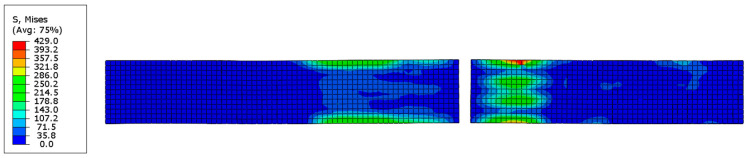
Overall stress distribution of the model.

After the calculations, the overall stress distribution of the CFRP model is shown in Figure 3. The middle section of the elements satisfies the damage criterion defined in the VUMAT subroutine, and the fully damaged elements are deleted.

**Figure 4:**
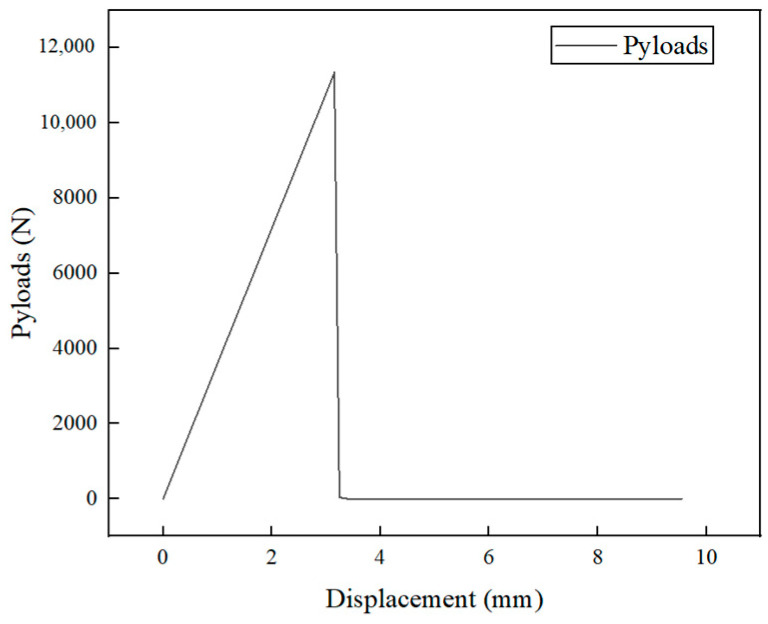
Load–displacement curve of the tensile fracture process of the model.

The load–displacement curve during the tensile process of the CFRP laminated plate is shown in Figure 4. The curve exhibits a clear linear characteristic, and after reaching the ultimate load, the material immediately loses its load-bearing capacity and undergoes brittle fracture, resulting in overall failure. The displacement at the peak load is 3.15 mm, and the ultimate load is 11,342 N.

Since the CFRP laminated plate has structural symmetry, we select the average strain changes between the first 10 layers to analyze and understand the response characteristics of the plate during the loading process.

**Figure 5:**
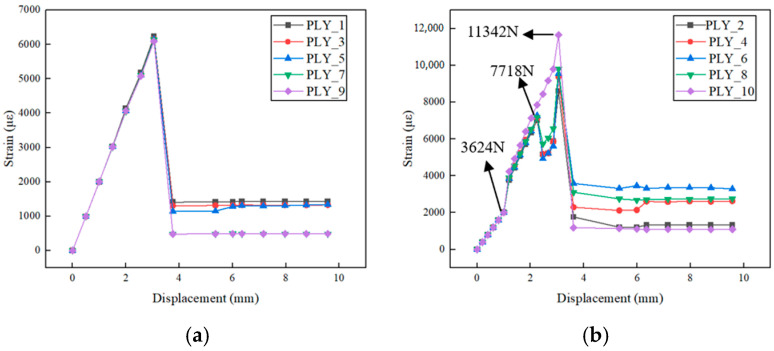
Interlayer strain variation during tensile loading: (**a**) strain in 0° lamination layer; (**b**) strain in 90° lamination layer.

Figure 5a shows that the strain in the 0° lamination layer increases linearly with the displacement load and decreases sharply after reaching the limit. The strain–displacement curve of the 0° lamination layer is consistent with the overall load–displacement curve, indicating that the 0° lamination fibers dominate the mechanical response of the material during tensile loading. Due to the high strength and modulus of carbon fibers, the material undergoes brittle fracture when the fiber strength limit is reached.

Figure 5b shows the strain changes in the 90° laminations. The figure indicates that although the strain in each layer increases with the load, the relationship gradually deviates from linearity. This suggests that with the increasing load, internal damage begins in the 90° fiber layer, leading to nonlinear strain variations. Below, we analyze the damage variables to explore the relationship between interlayer strain changes and damage evolution in the CFRP structure.

**Figure 6:**
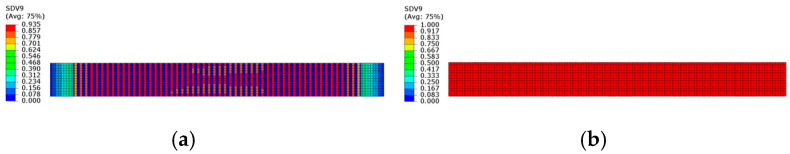
Matrix tensile damage in the 90° sub-layer: (**a**) F = 3624 N; (**b**) F = 7718 N.

**Figure 7:**
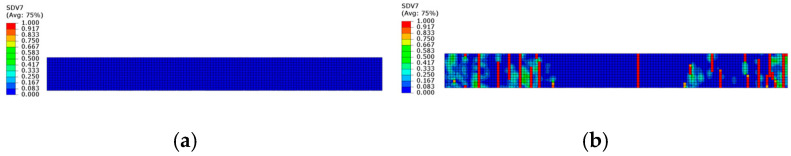
Fiber tensile damage in the 0° sub-layer: (**a**) F = 7718 N; (**b**) F = 11,432 N.

In this study, the damage propagation of the same layers in the orthogonal laminate was similar. Therefore, the first layer (0° ply) and the second layer (90° ply) were selected to examine the damage. The main damage type in the 0° sub-layer was fiber tensile damage, while the 90° sub-layer primarily exhibited matrix tensile damage. The damage variation in the simulation process is shown in Figure 6 and Figure 7:

At the first stage, within the load range of 3624 N, the strain in all layers exhibited a linear upward trend with the increasing load, indicating that the material’s internal condition remained good.

In the second stage, between 3624 N and 7718 N, the strain increased sharply with the load at approximately 3624 N, and the rate of strain change with load also increased. The reason is that the matrix has a relatively low tensile strength. Microcracks and plastic deformation accumulated in the matrix, causing matrix tensile failure in the middle region of the sub-layer. The damage led to a reduction in matrix tensile capacity, changing the local stiffness of the material and resulting in strain concentration, which accelerated the strain increase with load.

In the third stage, between 7718 N and 11,342 N, the strain in the 2nd, 4th, 6th, and 8th layers initially decreased and then increased with load, while the strain in the 10th layer continued to increase with load. At around 7718 N, the matrix in the 2nd, 4th, 6th, and 8th layers completely failed, with the damage index reaching 1, causing the strain to decrease rapidly. Stress and strain redistributed, and since the matrix in the 10th layer had not fully failed, the strain continued to increase with the load.

Finally, at around 11,432 N, the fibers in the 0° ply layer experienced tensile failure after bearing a large amount of tensile load. The fiber breakage caused the entire structure of the laminated plate to fail, resulting in the material cracking and losing its load-bearing capacity.

By conducting a tensile failure simulation of CFRP laminated plates, the evolution process of internal structural damage was revealed, and a corresponding relationship between internal damage variation and interlayer strain variation was established. By monitoring the internal strain variation in the laminated plate, internal damage can be effectively monitored, and preliminary judgments about damage types can be made. The high accuracy and sensitivity of FBG sensors enable the precise capture of strain variation in the material. Therefore, an experiment was designed to embed FBG sensors into locations in the CFRP laminated plate prone to damage, allowing for effective monitoring and evaluation of internal structural damage.

### 3.3. Finite Element Simulation Analysis of High-Temperature Decomposition

Given that aviation thermal insulation materials are often exposed to high-temperature environments, a high-temperature thermal decomposition simulation analysis was conducted using ABAQUS to further explore the internal structural changes under these conditions. Through numerical simulation, the thermal decomposition behavior and damage evolution process of the material under high temperatures were studied in detail to provide reference for subsequent experiments.

**Figure 8:**
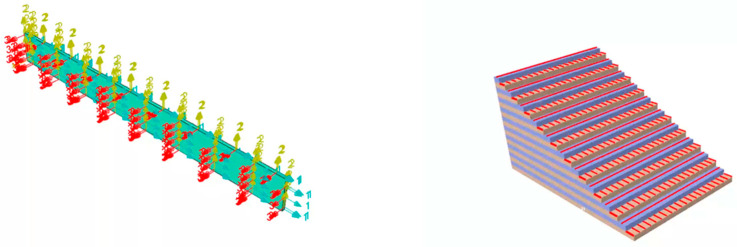
Lamination sequence of CFRP laminated plate.

A 3D solid model with dimensions of 250 × 25 × 2.5 mm was established in ABAQUS. The entity is divided into 20 pieces along the thickness direction, and 0°/90° alternating layer laying is achieved by assigning material directions. After the laying is completed, the interlayer stacking of the test piece is shown in Figure 8, which is the same as Figure 1. The mesh was generated by first applying global seeds of 0.005, and then assigning local seeds of 10 in the thickness direction. The element type used was the thermal transmission DC3D8 element. The surface heat exchange coefficient was set to 15, the surface radiation emissivity was set to 1, and the environmental temperature was set to 650 °C. A predefined temperature field of 25 °C was applied to the entire model.

**Figure 9:**
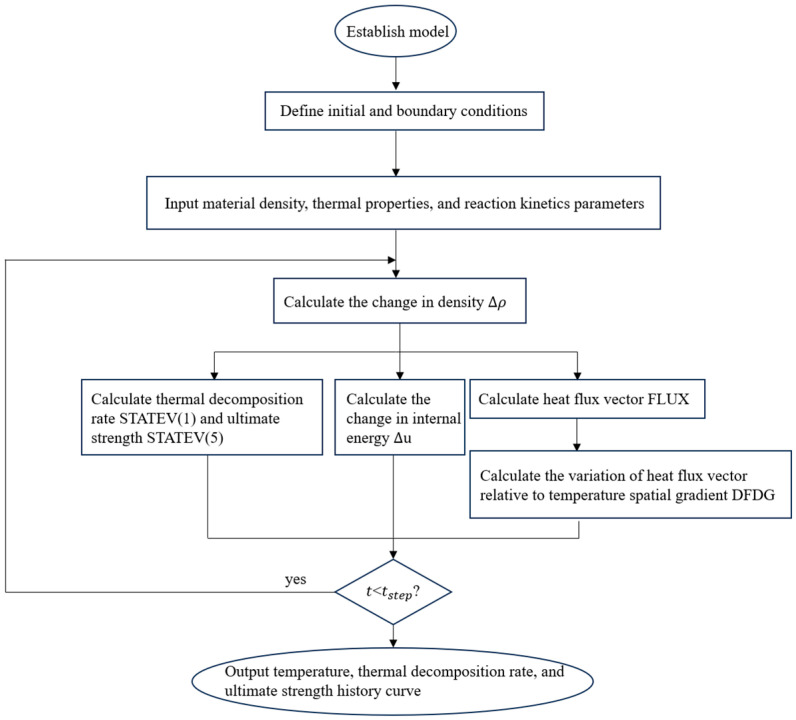
Subroutine calculation flow chart.

Based on the 1D transient thermal model for composites proposed by Henderson et al. [19], and the equation for predicting the ultimate strength of CFRP plates calibrated by Ke Wang et al. [20], the UMATHT subroutine was developed in FORTRAN for secondary development in ABAQUS. The calculation flow of the UMATHT subroutine is shown in Figure 9. The thermal property parameters used are the thermal decomposition rate, anisotropic thermal conductivity, and specific heat capacity of the solid material.

The thermal decomposition rate is expressed as follows:

(12) $$F=\frac{\rho_{v}-\rho }{\rho_{v}-\rho_{d}}$$

where $\rho_{v}$ represents the density of the original material, $\rho_{d}$ represents the density of the carbonized material, and ρ represents the instantaneous material density. A thermal decomposition rate of 0 indicates that the material is in its original state and has not undergone pyrolysis, while a rate of 1 indicates that the material is fully carbonized and completely pyrolyzed.

The expression for anisotropic thermal conductivity is as follows:

(13) $$k_{i}=\left(1-F\right)k_{vi}+Fk_{di}(i=x,y,z)$$

where $k_{vi}$ and $k_{di}(i=x,y,z)$ are the thermal conductivity of the raw material and the residual material after pyrolysis reaction, respectively, and F is the thermal decomposition rate.

The specific heat capacity of solid materials can be expressed as

(14) $$C_{p}=\left(1-F\right)C_{pv}+FC_{pd}$$

where $C_{pv}$ is the specific heat capacity of the raw material, $C_{pd}$ is the specific heat capacity of the residual material after pyrolysis reaction, and F is the thermal decomposition rate.

Chen and Young [21] proposed a model to describe the change in ultimate strength of materials exposed to high temperatures:

(15) $$\frac{f_{u,T}}{f_{u,normal}}=A-\frac{{\left(T-B\right)}^{n}}{C}$$

$f_{u,T}$ represents the ultimate strength of the material at temperature T, and $f_{u,normal}$ represents the ultimate strength of the material under normal conditinos. In Ke Wang [20], the coefficients A, B, C, and n were calibrated to align with experimental results for carbon fiber composites at a high temperature of 706 °C, which is close to the experimental temperatures in this study. Therefore, this model is utilized in the development of subroutines to predict changes in ultimate strength.

### 3.4. Simulation Results and Analysis

**Figure 10:**
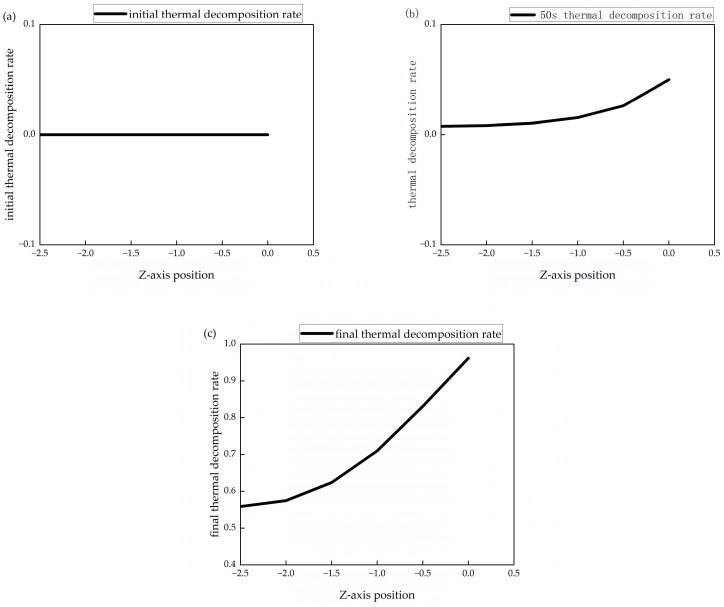
Thermal decomposition rate of model: (**a**) initial thermal decomposition rate; (**b**) 50 s thermal decomposition rate; (**c**) final thermal decomposition rate.

Figure 10 shows the thermal decomposition rate curves along the thickness direction of the material at the initial moment, 50 s, and the final moment under an environmental temperature of 650 °C. Here, Z = 0 mm represents the heated surface of the material, while Z = −2.5 mm represents the backside surface. The thermal decomposition rate reflects the degree of thermal decomposition reactions in the material, where a higher thermal decomposition rate indicates a more complete decomposition reaction, leading to a greater change in material density and a more significant reduction in the resin matrix. As the simulation progresses, the longer the heating time, the higher the material’s thermal decomposition rate. Analysis indicates that the heated surface of the material is affected by external heat flux, causing the temperature of the heated surface to rise the fastest and reach the pyrolysis temperature the earliest, resulting in the most complete pyrolysis. At the same moment, the closer to the Z = 0 mm surface, the higher the thermal decomposition rate.

**Figure 11:**
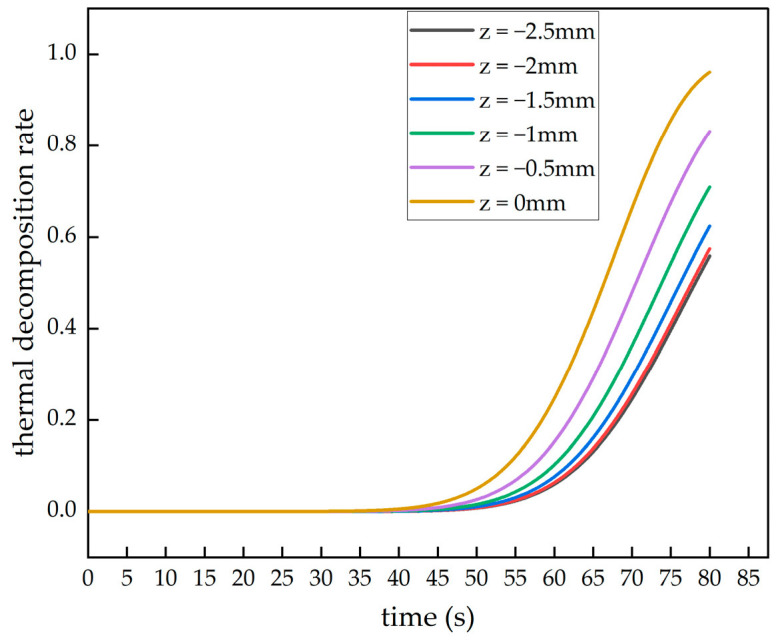
Thermal decomposition rate changes with time at different locations.

From the heated surface of the laminated plate, six surfaces were evenly selected along the negative Z-axis, with 0 mm representing the heated surface and −2.5 mm representing the unheated back surface of the material. The thermal decomposition rate data during the heating process for these six surfaces is shown in Figure 11. As observed in Figure 11, during the first 40 s of heating, since the material temperature did not yet reach the critical thermal degradation temperature, no thermal decomposition reaction occurred. After 40 s, as the temperature further increased, the heated surface of the laminated plate first reached the critical thermal decomposition temperature and began the thermal decomposition reaction. Subsequently, the other surfaces gradually reached the critical thermal decomposition temperature and started to degrade. By the end of the simulation, the thermal decomposition rate at the heated surface of the laminate reached 0.96. This rate indicates that the material density at the heated surface is very close to that of fully decomposed material, suggesting almost complete thermal decomposition. Conversely, the thermal decomposition rate was found to be the lowest at 0.56 at the z = −2.5 mm surface.

**Figure 12:**
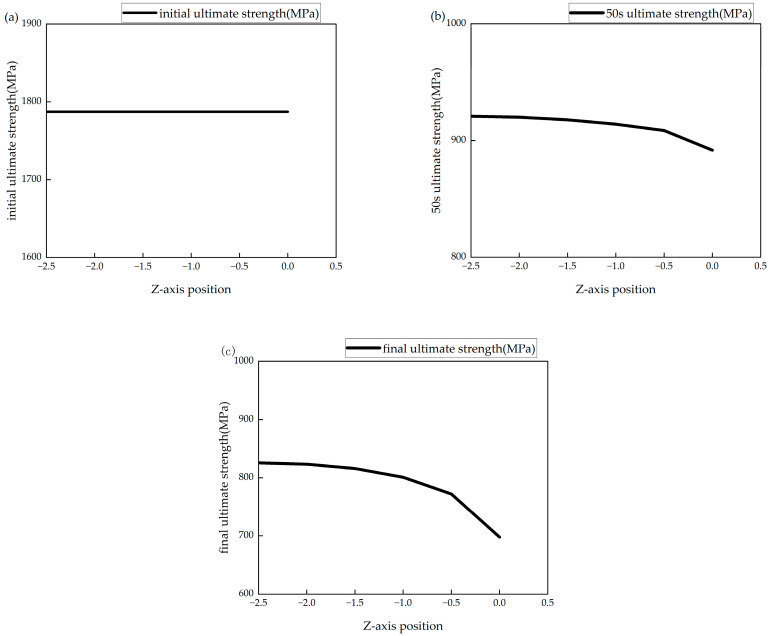
Ultimate strength of model: (**a**) initial ultimate strength; (**b**) 50 s ultimate strength; (**c**) end ultimate strength.

**Figure 13:**
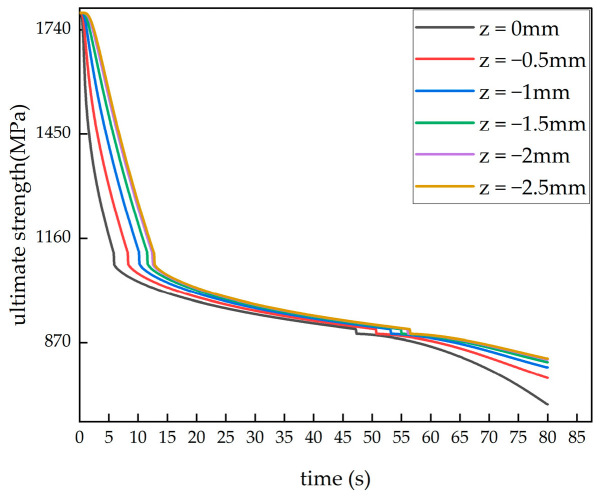
Ultimate strength changes over time at different locations.

Figure 12 shows the variation in the ultimate strength of the model with the material thickness direction at the initial moment, 50 s and the final moment under the ambient temperature of 650 °C. At the beginning of the simulation, the laminated plate had not yet been affected by the heat flow, and the ultimate strength of each surface was at its initial value. As the simulation progressed, at 50 s, the ultimate strength of the heated surface decreased to 50% of its original value, while the surface at −2.5 mm had decreased to 51.6% of its original value. By the end of the simulation, the ultimate strength of the heated surface had decreased to 40%, while the surface at −2.5 mm had decreased to 46%. The rate of ultimate strength reduction varied at different locations, with surfaces farther from the heated side requiring more time to reach the same level of strength reduction. Figure 13 shows the ultimate strength changes over time at different locations in the CFRP laminated plate under a 650 °C single-sided heating condition.

By combining Figure 11 and Figure 13, we can explore the reasons for the performance degradation of carbon fiber-reinforced composite materials (CFRPs) under high temperatures. During the first 40 s of the simulation, the thermal degradation reaction in the laminated plate matrix had not yet started. Therefore, the performance degradation at this stage was not caused by matrix degradation, but rather due to the softening of the epoxy resin matrix when the temperature gradually reached its glass transition temperature. The softening of the epoxy resin led to a gradual loss of its original strength and stiffness. After 40 s, the thermal degradation reaction in the laminated plate matrix began, and the carbon fibers lost support from the matrix, rendering them unable to effectively transfer stress. This further caused a reduction in the material’s ultimate strength. As mentioned in Wang’s study [20], when the temperature increased above 600 °C, carbon fibers began to oxidize. Although carbon fibers are resistant to high temperatures, they undergo chemical reactions at this temperature, generating carbon monoxide (CO) and carbon dioxide (CO₂), leading to the gradual consumption of carbon fibers and the loss of their load-bearing capacity. This further decreased the ultimate strength of the material.

## 4. Experiment and Analysis

**Figure 14:**
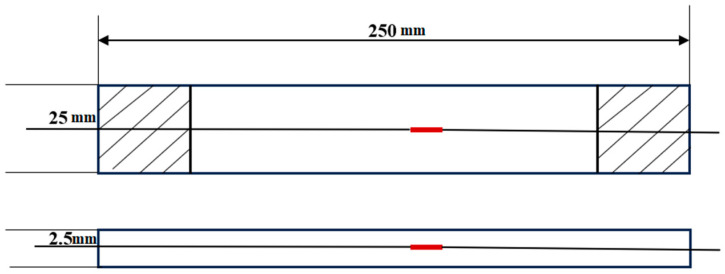
Dimensions of the specimen.

Based on the materials used in aviation thermal insulation layers, this study used T300/M03 carbon fiber prepreg to fabricate CFRP laminated plates, with a ply sequence of [0/90]_20s_, and each ply had a thickness of 0.125 mm, with a total thickness of 2.5 mm. The FBG sensor used is a single bare optical fiber. It should be noted that the temperature can reach 650 °C in the subsequent test, and the fiber coating layer cannot withstand this high temperature. Therefore, the coating layer should be removed before burying the fiber. Before curing, place the bare optical fiber in the corresponding layer and cure it together with the composite material. The curing process uses RTM technology and high-temperature curing. The specimens were fabricated according to the recommended tensile specimen geometry in ASTM D3039. FBG sensors were embedded inside the CFRP specimens, with sufficient protection for the internal and exposed ends of the sensors to ensure the feasibility and reliability of embedding sensors into the composite materials. To minimize the structural impact of the embedded fiber, the total thickness of composite laminates was designed to be more than ten times the diameter of the bare fiber, and the fiber was drawn out from the side of the composite material. The structure and dimensions of the CFRP laminated plates are shown in Figure 14. The red rectangle in Figure 14 represents the FBG sensor.

In this experiment, two CFRP laminates embedded with FBG sensors and one CFRP laminate without FBG sensors were designed and prepared, referred to as specimens 1, 2, and 3, respectively. The two CFRP laminates embedded with FBG sensors share the same materials, dimensions, ply angles, number of layers, and fiber embedding techniques. The dimensions and shapes of specimens 1 and 2 are shown in Figure 14.

Specimen 3, while not embedded with FBG sensors, has identical conditions to specimens 1 and 2 in all other aspects. According to Zhou et al. [22], the tensile strength of composite laminates embedded with fiber Bragg gratings in symmetric internal layers is reduced by 5.36% compared to those without embedded gratings, indicating minimal impact. To verify whether embedding FBGs in this experiment results in a similar effect on the strength of laminates, a tensile fracture test was designed for specimen 3.

In order to simulate the complex load environment during the use of CFRP laminates as aviation insulation layers and explore the ability of FBG sensors to monitor the internal state of aviation insulation layers in real-time, stably, and for a long time, tensile fracture tests were conducted on specimen 1, and high-temperature sintering and tensile fracture tests were conducted on specimen 2. To ensure the standardization and comparability of the experiments, all tensile tests were performed according to ASTM D3039 [23].

### 4.1. Tensile Fracture Test for Specimen 1

This experiment aimed to simulate the working conditions of CFRP laminated plates under axial loads without temperature influence, mimicking their performance on the ground. By performing long-term axial loading experiments, the internal structural changes in aviation thermal insulation materials were studied, and the real-time monitoring capability of FBG sensors under these conditions was further validated.

**Figure 15:**
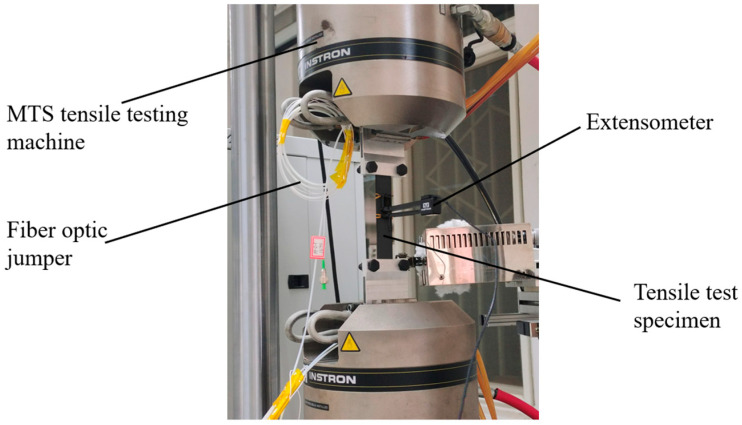
Tensile testing system diagram.

A static test was conducted on the INSTRON-8801(manufacturer: INSTRON, equipment source: Shanghai, China) testing machine in a room temperature dry condition of 18 °C. The prepared CFRP specimen 1 was placed in the grips of the Instron 8801 testing machine and securely fixed to ensure the alignment of the grips with the central axis, thereby avoiding uneven stress distribution due to eccentric tension, which could increase the risk of premature specimen failure. The experimental setup is shown in Figure 15. During the test, a demodulator was used to decode the wavelength signals from the sensor, with a demodulation frequency of 1 Hz.

First, a calibration test for specimen 1 was conducted. The axial load was increased in steps of 500 N, with each load step held for 30 s to reach a stable state. The load was increased to 3 kN and then unloaded in steps, repeating the process three times. The strain values measured by the extensometer and the fiber sensor were recorded in real-time during the calibration test of specimen 1. Then, a tensile fracture test was performed, with the loading rate controlled at 1.0 mm/min by displacement. After the test, the load and deformation data were recorded, and the failure mode of the specimen was observed.

#### 4.1.1. Calibration Data Processing for Specimen 1

**Figure 16:**
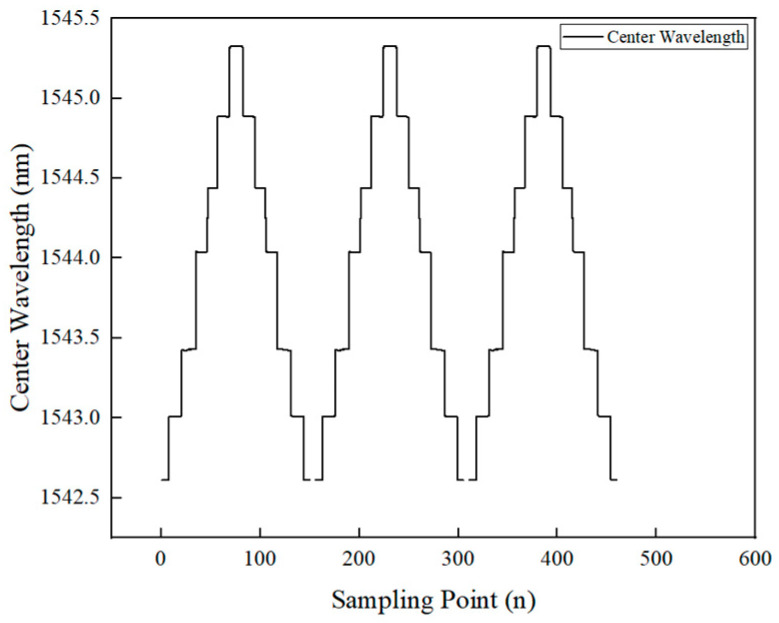
Calibration data for specimen 1.

The wavelength variation data of the FBG sensor during the calibration test for CFRP specimen 1 is shown in Figure 16.

**Figure 17:**
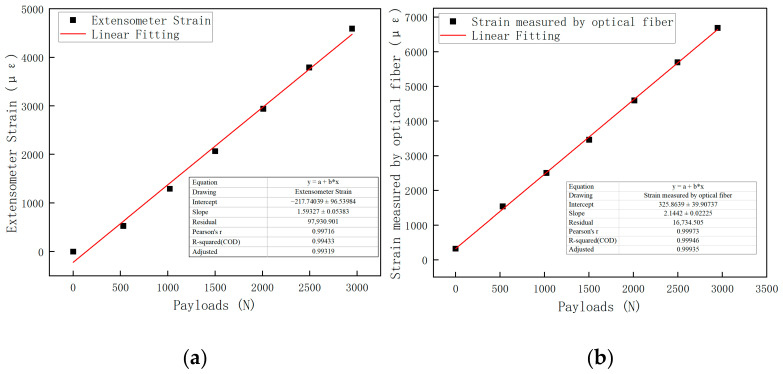
Load–strain fitting curves: (**a**) extensometer; (**b**) optical fiber.

As shown in Figure 16, the FBG wavelength data from the three repeated tests were almost identical, demonstrating the excellent stability and reliability of the FBG sensor. The average strain values from the extensometer and the fiber sensor at each load level were calculated from the first cycle and correlated with the corresponding load for analysis. The load–strain statistics for each load level and the fitted curves for the extensometer and fiber sensor are shown in Figure 17a,b.

As shown in Figure 17, within the 3 kN range, the external strain measured by the extensometer exhibits a linear relationship with the axial load, with a linearity of 0.997. This indicates that the surface of the CFRP specimen undergoes elastic deformation within this range. Similarly, the internal strain of the specimen measured by the optical fiber also shows a linear relationship with the axial load, with a linearity of 0.999. The fitted equation is y = 2.14x + 325.86. This suggests that within the 3 kN range, the internal structure of CFRP specimen 1 remains intact, exhibiting elastic deformation behavior. The sensitivity of strain to the external load is 2.14 με/N. Table 2 is the Load–strain statistics.

It is worth noting that the extensometer is placed on the surface of the specimen, so it measures the surface strain, while the optical fiber is embedded inside the specimen and measures the internal strain. Due to the laminate structure of the specimen, there is an attenuation of stress transfer between layers. Therefore, it is normal for the strains measured by the extensometer and the optical fiber to differ, and a nonlinear relationship between them is expected. At this stage, it is demonstrated that the embedded FBG sensor can monitor the internal strain of the CFRP specimen in real-time and establish correspondence between strain and load under normal conditions, providing a reference for subsequent experiments.

#### 4.1.2. Tensile Fracture Test Data Processing for Specimen 1

**Figure 18:**
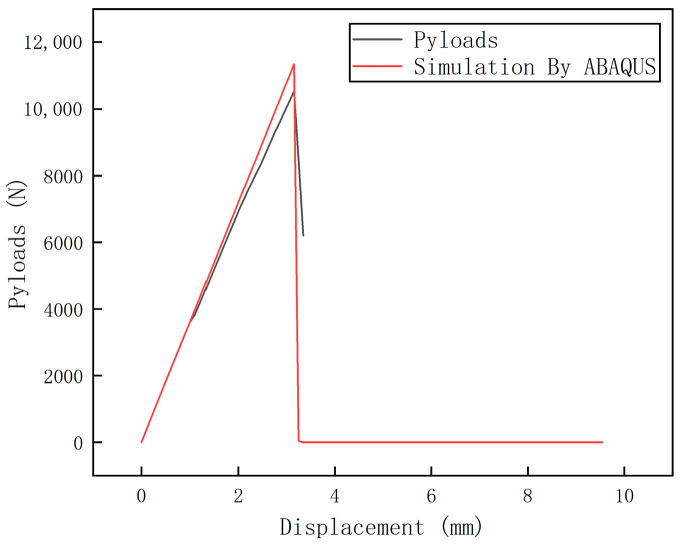
Comparison between numerical calculation and experimental results.

The load–displacement curve obtained from the tensile test and the finite element simulation results are compared in Figure 18.

As shown in Figure 18, the ultimate load predicted by the finite element simulation was 11,432 N, which is 902 N higher than the experimental result of 10,530 N, yielding a relative error of 8.5%. This indicates that the finite element prediction of ultimate strength is reliable.

**Figure 19:**
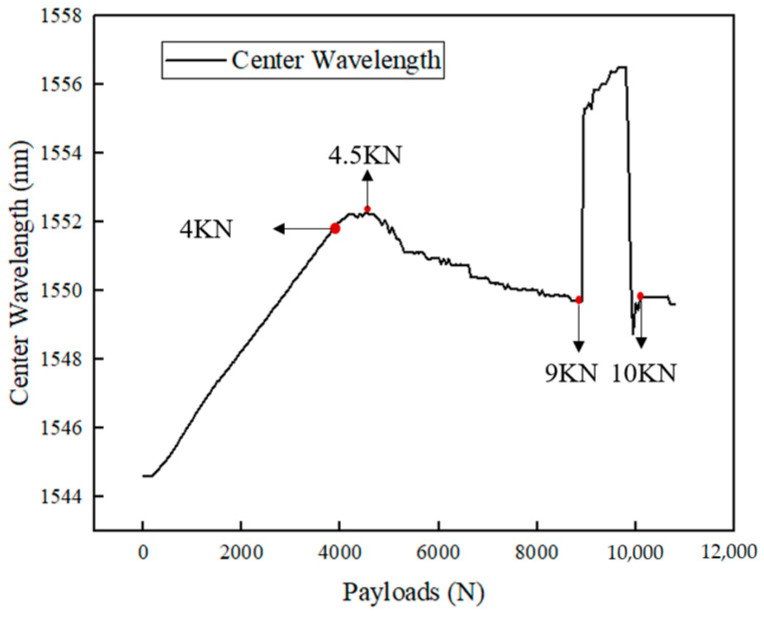
Wavelength of the FBG sensor during tensile testing.

The wavelength shift in the FBG sensor during the tensile test is shown in Figure 19.

As shown in Figure 19, within the 4 kN range, the wavelength increased linearly with the load, indicating elastic deformation, and the internal structure of the CFRP remained in good condition. This demonstrates that the FBG sensor could normally monitor the internal structure of the CFRP.

Between 4 kN and 4.5 kN, the wavelength exhibited an overall fluctuating upward trend with the increase in load, and the linear relationship disappeared. Based on the finite element analysis, it is inferred that at this stage, the matrix in the 90° plies began to experience tensile damage, and microcracks started to form, leading to uneven strain distribution inside the material. The FBG sensor accurately captured this non-uniformity and the initial damage in the material.

From 4.5 kN to 9 kN, the wavelength of the FBG sensor began to decrease as the load increased, indicating that internal damage was gradually accumulating, and that the structure was progressively deteriorating. Damage in the matrix near the FBG sensor caused delamination between the plies, leading to local strain release and a nonlinear strain distribution. The wavelength changes recorded by FBG sensors reflect the impact of these local damages on the overall structure, and the fluctuating strain curve also indicates that the damage is expanding, affecting the area near the FBG sensor.

**Figure 20:**
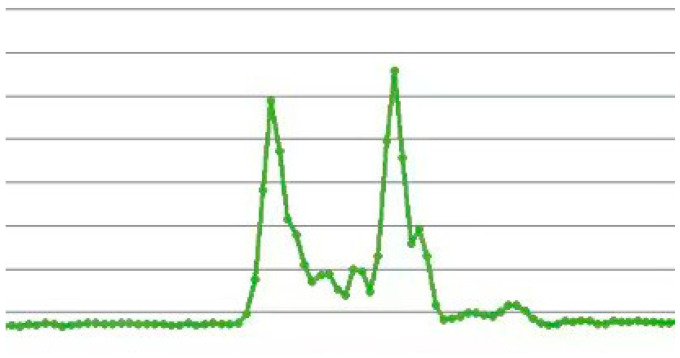
Chirping phenomenon of the FBG waveform.

Around 9 kN, the FBG waveform exhibited chirping, in the case of only bearing axial loads, the influence of transversal strain is not sufficient to cause chirping in the waveform. Therefore, it is believed that fibers in the 0° ply near the FBG sensor began to experience tensile failure, generating shear forces that compressed the FBG sensor’s grating area. The FBG waveform chirping is shown in Figure 20. The demodulation software displayed the original waveform splitting into two, which caused a significant increase in the recorded wavelength value. This reflects the drastic changes that occur when the FBG sensor gate area is squeezed.

Finally, at around 10 kN, the material reached its load-bearing limit. The 0° ply fibers experienced massive tensile failure, leading to the total failure of the laminated plate, with the material cracking and losing its entire load-bearing capacity.

**Figure 21:**
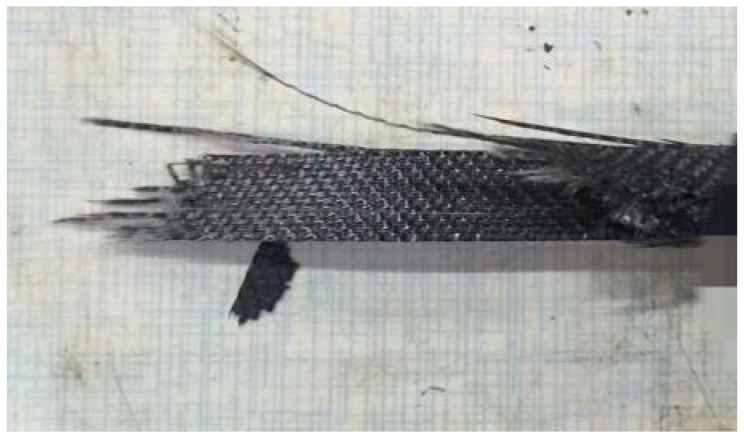
Fracture of the specimen.

The failure of the specimen is shown in Figure 21, where a sound was heard as the sample neared fracture. Fibers in the 0° layer were pulled out and fractured in large quantities. The fracture occurred mainly in the middle of the specimen, and the primary failure modes were fiber pull-out and matrix damage, consistent with the results of the finite element simulation.

In summary, from the initial elastic deformation stage to damage accumulation and expansion, and finally to complete fracture failure, the FBG sensor was able to precisely capture strain changes caused by internal micro-damage in the material. This demonstrates that the FBG sensor, through the real-time monitoring of wavelength shifts, can effectively detect internal damage in aviation thermal insulation materials and accurately reflect microstructural changes under tensile load. The sensor sensitively detected damage initiation and the evolution process, providing a preliminary assessment of the type of damage. This provides reliable data support for structural health monitoring and early damage warning in aviation thermal insulation materials.

#### 4.1.3. Comparison of Ultimate Strength Between Specimen 3 and Specimen 1

Specimen 3, which does not contain embedded FBG sensors, does not require calibration testing, and was directly subjected to tensile failure testing. The experiment was conducted using an INSTRON-8801 testing machine under ambient conditions at 18 °C in a dry state. The prepared CFRP specimen 3 was placed in the grips of the Instron 8801 testing machine and firmly secured to ensure the alignment of the grips with the central axis, thereby preventing uneven stress distribution caused by eccentric loading, which could increase the risk of premature failure.

**Figure 22:**
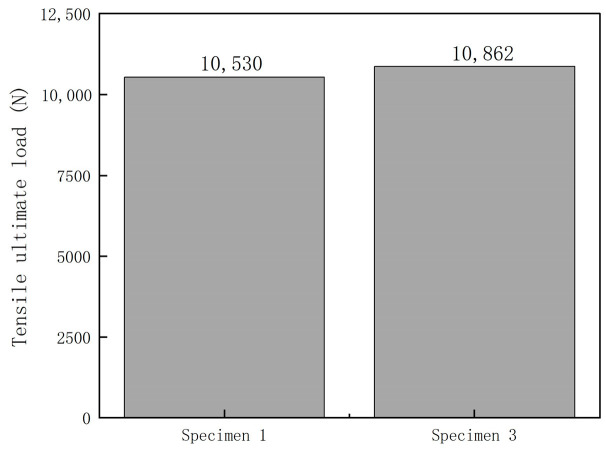
The impact of embedded fiber Bragg gratings on the tensile strength of laminate specimens.

Figure 22 presents the test results for the ultimate loads of specimens 1 and 3. From the results, it can be observed that the ultimate tensile load of specimen 1 was 10,530 N, whereas that of specimen 3 was 10,862 N. The tensile ultimate load of the composite laminate embedded with fiber Bragg gratings in the center-symmetric layer decreased by 3.06% compared to the laminate without embedded gratings.

### 4.2. High-Temperature Sintering Experiment of Specimen 2

**Figure 23:**
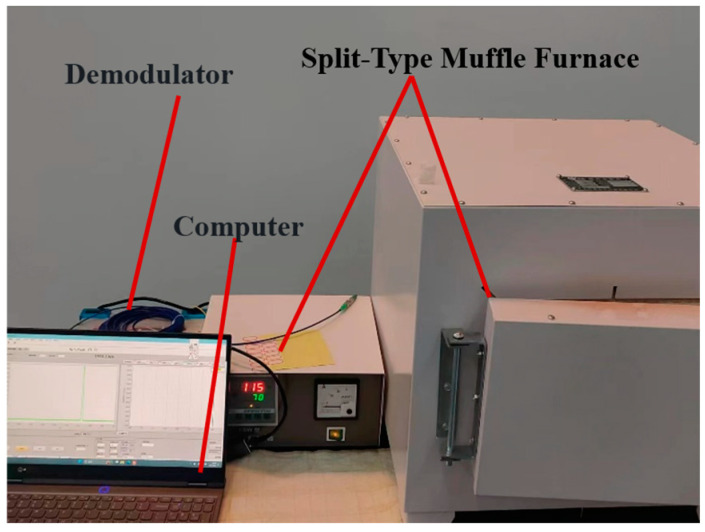
High-temperature sintering experimental setup.

Aerospace thermal insulation materials typically serve in extreme high-temperature environments, where prolonged exposure to such conditions can adversely affect the mechanical properties, structural stability, and lifespan of the material. This experiment aims to study whether the fiber Bragg grating embedded in aerospace thermal insulation materials can continue to provide stable monitoring in these environments. A split-type SX2-5–12 muffle furnace is used to perform high-temperature sintering on test specimen 2. Starting from room temperature, the initial set temperature is 400 °C. Once the temperature stabilizes, the set temperature is raised, and this process is repeated 10 times. The temperature is increased to 650 °C before stopping the experiment. The FBG sensor is used to dynamically monitor the strain changes inside the material in real time throughout the process. The center wavelength changes in the FBG during sintering are recorded using a PC. The experimental setup is shown in Figure 23.

#### High-Temperature Sintering Experimental Data Processing

**Figure 24:**
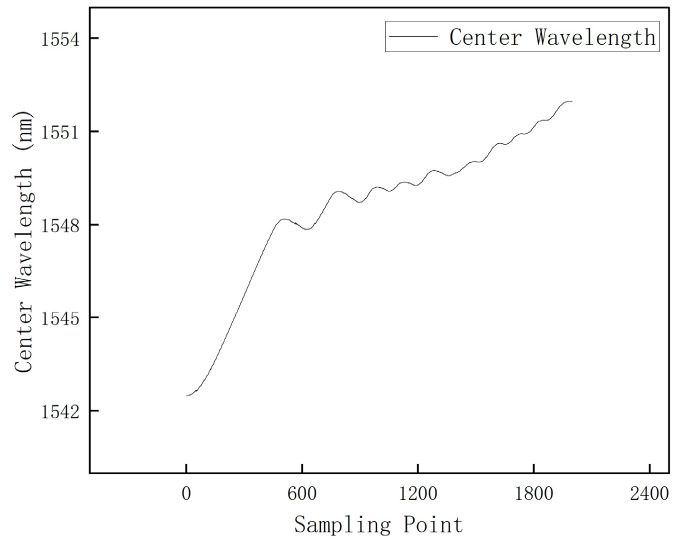
High-temperature sintering wavelength variation diagram for specimen 2.

The wavelength variation in the FBG sensor during the high-temperature experiment is shown in Figure 24. From Figure 24, it can be seen that during the process of heating up to 650 °C, FBG stably monitored the changes throughout the entire process, indicating that FBG can work stably in extremely high-temperature environments.

### 4.3. Tensile Fracture Test for Specimen 2 After High-Temperature Treatment

**Figure 25:**
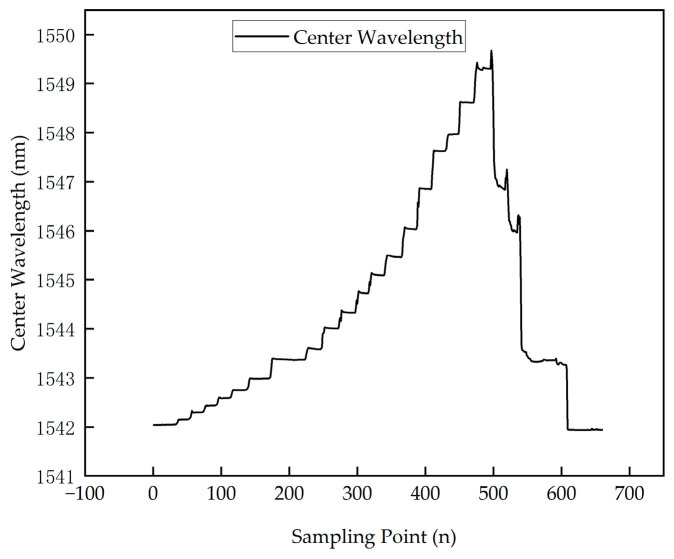
Tensile fracture experiment for specimen 2.

The high-temperature sintering experiment verified that the FBG can still function normally for monitoring in high-temperature environments. To investigate whether the FBG can continue to stably monitor the internal structural state of the material after high-temperature treatment, a tensile failure test was conducted on cooled test specimen 2. The experiment used the same tensile test system as test specimen 1, and specimen 2 was mounted on the fixture. Since the mechanical properties of the carbon fiber composite laminate after high-temperature burning are unclear, a stepwise stable load was applied to the specimen until material failure and fracture occurred. After organizing the data measured by the optical fiber, the strain data measured from the high-temperature burning test specimen was obtained, as shown in Figure 25. Compared with the tensile test of test specimen 1, it was found that after high-temperature treatment, the tensile properties of the CFRP laminate decreased significantly. It can withstand 8KN of support under normal conditions, but brittle fracture occurred at 2KN after high-temperature treatment. Growth intensity decreased by 75%.

As shown in Figure 25, despite undergoing high-temperature treatment, the FBG sensor structure and performance were not irreversibly damaged, and the sensor was still able to monitor the mechanical performance of the specimen, continuing to measure strain in real-time. This demonstrates the stability and reliability of the FBG sensor in high-temperature environments and its ability to monitor the structural health of CFRP materials under complex conditions.

**Figure 26:**
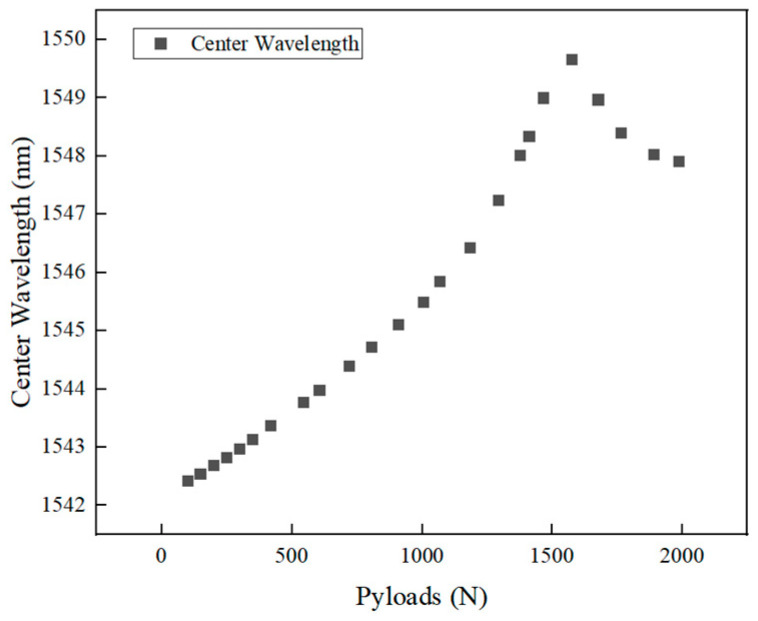
Wavelength changes in specimen 2 during tensile fracture testing.

The scatter plot in Figure 26 was drawn by selecting the average wavelength values of the FBG sensor at each stable load step.

As shown in Figure 26, within the initial 1 kN load range, despite the high-temperature treatment, the strain measured by the FBG sensor still showed a good linear relationship with the load. This indicates that at low loads, the overall stiffness and integrity of the material had not completely degraded, and the CFRP material still exhibited elastic deformation.

When the load exceeded 1 kN, the strain–load relationship began to deviate from linearity, and the FBG sensor displayed strain values that increased more significantly than the load. This nonlinear response suggests that internal damage had gradually accumulated, particularly as the resin’s evaporation during high-temperature treatment weakened the bond between fibers and the matrix. This stress concentration at the interface further triggered matrix cracking, microcrack propagation, and interlayer delamination damage mechanisms. At this stage, the stress distribution in the material became uneven, and the internal structural damage slowly expanded.

When the load exceeded 1.5 kN, despite the continued increase in external load, the FBG wavelength began to decrease. This indicates that internal damage accumulation had accelerated, and the micro-damage caused by high-temperature treatment further spread, reducing the interfacial stress transfer efficiency between fibers and the matrix. Fiber bundles may have begun to slip or debond. The local stiffness gradually decreased, explaining the observed strain drop.

At around 2 kN, specimen 2 reached its ultimate failure, with damage accumulation leading to brittle fracture. Compared to specimen 1, which was not exposed to high-temperature treatment, specimen 2 exhibited early failure at a much lower load due to the evaporation of the resin, which caused significant microcracks and interface damage, making the material unable to bear higher loads. The FBG sensor accurately captured the process of damage accumulation and expansion, further confirming its sensitivity and effectiveness in monitoring internal strain changes and damage evolution.

The tensile test results for specimen 2 after high-temperature treatment showed that although significant internal changes occurred, the FBG sensor structure remained functional, and it could still monitor the strain of the material in real time. This highlights the stability and reliability of FBG sensors in high-temperature conditions.

## 5. Conclusions

This study employed finite element simulations and experiments on CFRP laminated plates embedded with FBG sensors to verify the effectiveness of FBG sensors in monitoring damage progression under complex stress and high-temperature conditions. The main conclusions are as follows:

- In CFRP laminated plates without high-temperature treatment, FBG sensors accurately captured the entire process from elastic deformation to final failure and provided preliminary identification of damage types. The damage evolution process, dynamically tracked through wavelength changes, was highly consistent with the finite element simulation results, validating the reliability of FBG sensors in complex stress environments.
- In CFRP laminated plates subjected to 650 °C high-temperature treatment, although the mechanical properties significantly deteriorated, with a 75% reduction in tensile strength, FBG sensors continued to effectively monitor internal strain changes, recording the entire progression from damage accumulation to final failure. This demonstrates the stability and durability of FBG sensors in high-temperature environments.

Although FBG sensors have stability and reliability in complex stress and high-temperature environments, there are still certain limitations as composite material structural health monitoring devices: firstly, calibration is required before use, secondly, temperature compensation is needed, and finally, there may be creep, and the long-term stability of the sensor needs to be verified.

Through this study, the long-term application potential of FBG sensors in monitoring aviation thermal insulation layers has been validated, providing important technical support for improving the safety and reliability of aircraft. Future research can focus on ident ifying the nonlinear relationship between internal strain and surface strain in laminated panels.

## Figures and Tables

**Table 1:** Material parameters of carbon fiber composite laminates.

Parameter	Value
ρ (kg/m^3^)	1700
E_1_/GPa	132
E_2_, E_3_/GPa	9.2
G_12_, G_13_/GPa	4.68
G_23_/GPa	3.35
μ_12_, μ_13_	0.312
μ_23_	0.32
X_T_/MPa	1622
X_C_/MPa	1270
Y_T_, Z_T_/MPa	46.2
Y_C_/Z_C_/MPa	179
S_12_, S_13_, S_23_/MPa	69

**Table 2:** Load–strain statistics.

Payloads/N	0	530	1021.800	1500.500	2009.390	2494.136	2946.701
Extensometer strain/με	0	529	1292.325	2066.714	2940.667	3790.483	4590.015
Strain measured by optical fiber/με	321.824	1538.721	2504.193	3459.608	4596.049	5692.262	6687.905

## Data Availability

Restrictions apply to the datasets. The dataset presented in this article is not readily available as the data are part of an ongoing follow-up study. For requests to access the dataset, please contact email: yanguang79@bistu.edu.cn.
